# Predictors of return to desired activity 12 months following unicompartmental and total knee arthroplasty

**DOI:** 10.1080/17453674.2018.1542214

**Published:** 2018-11-19

**Authors:** Alexander D Harbourne, Maria T Sanchez-Santos, Nigel K Arden, Stephanie R Filbay

**Affiliations:** a Nuffield Department of Orthopaedics, Rheumatology & Musculoskeletal Sciences, University of Oxford, Botnar Research Centre, Oxford, UK;;; b Arthritis Research UK Centre for Sport, Exercise and Osteoarthritis, Nuffield Department of Orthopaedics, Rheumatology & Musculoskeletal Sciences, University of Oxford, Botnar Research Centre, Oxford, UK;;; c MRC Lifecourse Epidemiology Unit, University of Southampton, UK

## Abstract

Background and purpose — 1 in 5 patients are dissatisfied following unicompartmental or total knee arthroplasty (UKA or TKA). This may be partly explained by failing to return to desired activity post-arthroplasty. To facilitate return to desired activity, a greater understanding of predictors of return to desired activity in UKA and TKA patients is needed. We compared rates of return to desired activity 12 months following UKA vs. TKA, and identified and compared predictors of return to desired activity 12 months following UKA vs. TKA.

Patients and methods — Patients were prospectively recruited from 2 hospitals prior to undergoing UKA or primary TKA. Patients reported preoperatively the activity/activities that were limited due to their knee that they wished to return to after arthroplasty. At 12-months postoperatively, patients reported whether they had returned to these activities (‘return to desired activity’). Preoperative predictors evaluated were age, sex, BMI, education, comorbidities, pain expectations, Oxford Knee Score (OKS), UCLA Activity Score, and EQ-5D. Generalized linear models assessed the relationship between potential predictors and return-to-desired-activity.

Results — The response rate of all patients eligible for 12-month follow-up was 74%. TKA patients (n = 575) were older (mean (SD) 70 (9) vs. 67 (10)) with a greater BMI (31 (6) vs. 30 (5)) than patients undergoing UKA (n = 420). 75% of UKA and 59% of TKA patients returned to desired activity. TKA patients had a greater risk of non-return to desired activity than patients undergoing UKA (risk ratio (95% CI) 1.5 (1.2–1.8)). Predictors of non-return to desired activity following UKA were worse OKS (0.96 (0.93–0.99)), higher BMI (1.04 (1.01–1.08)), and worse expectations (1.9 (1.2–2.8)). Predictors of non-return to desired activity following TKA were worse EQ-5D (0.53 (0.33–0.85)) and worse OKS (0.98 (0.96–1.0)).

Interpretation — UKA patients were more likely to return to desired activity than TKA patients. Predictors of return to desired activity differed following UKA and TKA. Optimizing selection of arthroplasty procedure based on patient characteristics and targeting predictors of poor outcome may facilitate return to desired activity with potential to enhance postoperative satisfaction.

As many as 20% of patients are dissatisfied with unicompartmental knee arthroplasty (UKA) (Von Keudell et al. 2014, Kim et al. 2017) and total knee arthroplasty (TKA) outcomes (Scott et al. 2010). This presents a significant concern considering that the number of both UKA and TKA procedures is rising in Europe and North America (Kurtz et al. 2007, Leskinen et al. 2012). 1 in 5 patients who receive TKA have isolated unicompartmental OA that could be treated by either procedure (Arno et al. 2011). Selecting the most appropriate knee arthroplasty procedure in line with a patient’s preoperative characteristics has the potential to improve postoperative satisfaction, function, and participation in desired activities. Selection of one surgical technique over the other is frequently based on traditional criteria, including that UKA patients should be over 60 years of age at the time of operation, not obese, not extremely physically active, have minimal knee pain at rest, and an adequate range of motion (Kozinn and Scott 1989, National Imaging Associates Inc. 2015). This may be due to a scarcity of literature that directly compares predictors of outcome following UKA and TKA procedures.

It is pertinent to assess postoperative outcomes that are of relevance and importance to patients. Patients who undergo UKA have a greater likelihood of returning to sport compared with TKA patients (Witjes et al. 2016). However, patients undergoing these procedures may desire participation in activities with contrasting demands, and it is not clear if a similar relationship exists with regards to returning to an individual’s desired activity. Improved function in desired activities that were limited preoperatively is expected by as many as 72% of knee arthroplasty patients (Nilsdotter et al. 2009). Failure to meet expectations regarding participation in desired activities is a key determinant of postoperative dissatisfaction following knee arthroplasty (Bourne et al. 2010, Scott et al. 2012). Identifying patient characteristics that predict return to desired activity following UKA and TKA may inform targeted preoperative interventions with potential to improve postoperative patient satisfaction. Therefore, we compared rates of return to desired activity 12 months following UKA vs. TKA, and identified and compared predictors of return to desired activity 12 months following UKA vs. TKA.

## Patients and methods

### The Clinical Outcomes in Arthroplasty Study (COASt)

The data for this analysis were collected through the Clinical Outcomes in Arthroplasty Study (COASt), a prospective, longitudinal cohort study based at 2 UK hospitals, Nuffield Orthopaedic Centre (NOC), Oxford and Southampton University Hospital NHS Foundation Trust (UHS) (Arden et al. 2017). Knee arthroplasty procedures were performed from 2010 to 2016. Patient-reported outcomes were collected at baseline (pre-operation) and at 12 months following knee arthroplasty.

#### Recruitment and procedure

Patients were recruited from waiting lists for hip or knee arthroplasty from both hospitals. To be eligible for COASt, patients were required to (i) be over 18 years of age at the time of recruitment, (ii) be competent and willing to consent to partake in the study, and (iii) not show signs of any severe neurological disorder.

A recruitment pack was sent to potentially eligible patients. Contact was subsequently made 2 weeks later to ascertain eligibility and a verbal desire to participate. If both were met, a research appointment was arranged to obtain baseline measurements and written consent. Baseline data were collected through questionnaires and a physical examination performed by a research nurse, physiotherapist, or podiatrist. Postoperatively, patients received follow-up questionnaires that were completed by post or during an appointment.

To be considered eligible for the current study, patients had to have undergone a UKA or primary TKA, and completed the 12-month follow-up questionnaire by post. Patients who had a hip arthroplasty, a revision TKA, or more than 1 arthroplasty on their knees were ineligible. As of May 2017, 1,491 individuals had undergone UKA or primary TKA. Patients who died (n = 36) or had a second UKA or TKA on either knee (n = 24) within the follow-up period were excluded (Figure). 1,431 patients were eligible to complete the 12-month follow-up questionnaire, of which 372 did not respond (response rate =74%). In addition, patients who completed the follow-up administered over the telephone (n = 38) and those who had missing data on the return to desired activity variable at baseline and/or 12-month follow-up (n = 26) were excluded. Data from 995 patients were included in this study (Figure). Indications for knee arthroplasty (not reported for 15% of participants) were OA (81%), rheumatoid arthritis (2%) and other reasons (2%).

**Figure:**
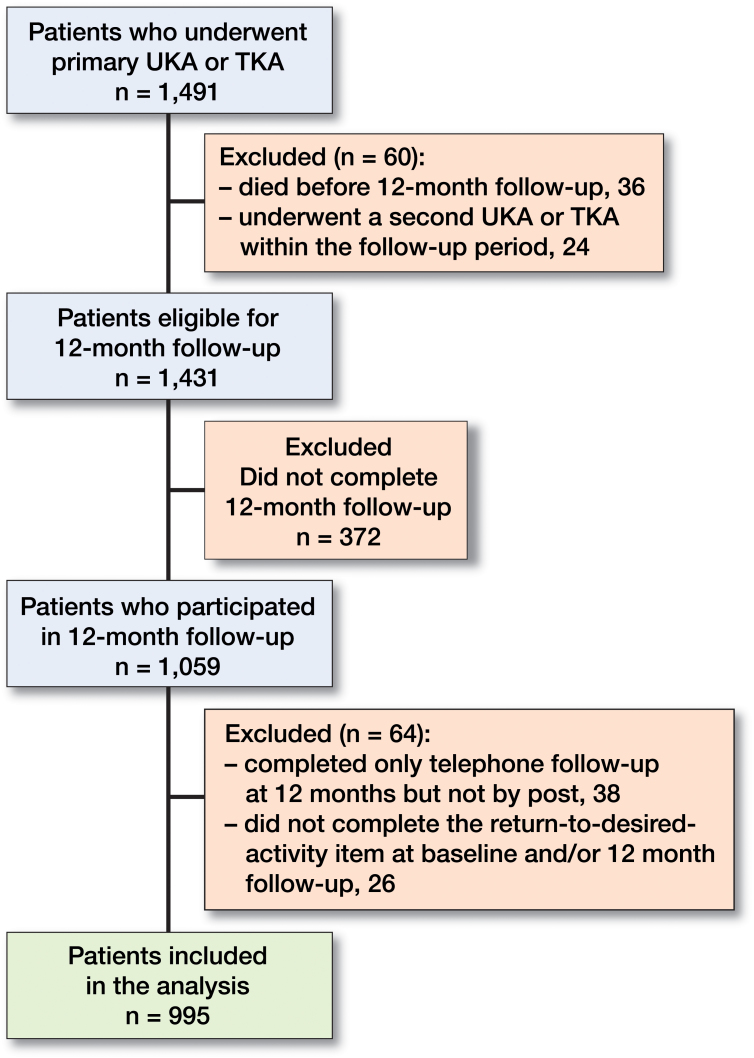
Participant recruitment flow chart. UKA: unicompartmental knee arthro­plasty; TKA: total knee arthroplasty.

#### Outcome—return to desired activity

Patients were asked at baseline: “What activity/activities does your knee stop or limit you from doing, that you wish to return to after your operation?” Patients were then given the option to report between 1 and 3 activities. Then at 12 months post-operation patients were asked: “Have you been able to return to the activity (or activities) that your knee stopped you from doing 12 months ago? (Yes/No).” Since not returning to desired activity (answering “No” to the above question) was the more infrequent outcome, predictors were assessed in relation to non-return to desired activity.

#### Potential predictor variables

Predictors used in this study were chosen following our review of the literature and clinical reasoning. To minimize over-adjustment and collider stratification biases, the selected variables were incorporated into directed acyclic graphs (DAGs; useful to depict assumed relationships between variables). They were performed in reference to a 6-step process to achieve unbiased estimates (Shrier and Platt 2008). The variables used in the DAGs are described below.

##### Patient demographics

Patient demographics included as potential predictors in this analysis were: the age at operation; sex; BMI; highest education level attained (dichotomized to “did not complete General Certificate of Secondary Education (GCSE) or above” and “completed GCSE or above”). The GCSE is a qualification in England, Wales, and Northern Ireland taken in a specific subject by school students typically aged 14–16 years.

###### Baseline Oxford Knee Score (OKS)

The OKS is a 12-item questionnaire designed to assess knee pain and function, with each item scored between 0 (worst outcome) and 4 (best outcome) (Dawson et al. 1998). Items are summated to give a total OKS score (possible range of 0–48) (Murray et al. 2007). A lower baseline OKS score suggests more pain and worse preoperative knee function. The OKS has adequate test–retest reliability, good sensitivity and responsiveness to change, and is valid for use in UKA and TKA populations (Collins et al. 2011, Jenny and Diesinger 2012). Mean substitutions were carried out in line with guidelines to replace missing items in OKS data (Murray et al. 2007).

###### University of California, Los Angeles Activity Score (UCLA-AS)

The UCLA-AS is a patient-reported measure that classifies a patient’s activity into 1 of 10 levels, where level 1 represents the lowest activity level (inactive and dependent on others) and level 10 represents the highest activity level (regular participation in contact sports). It is valid for clinical assessment of routine activity following knee arthroplasty (Zahiri et al. 1998, Fisher et al. 2006).

###### EuroQol-5D-3L (EQ-5D)

The EQ-5D is a self-reported measure of general health status. It comprises 5 questions relating to a patient’s mobility, self-care, usual activities, pain/discomfort, and anxiety/depression. Each has 3 levels of response and an overall 5-part profile is calculated using weightings outlined in EuroQol Group guidelines (EuroQol Group 1990). Index values (between –1 and 1) were then generated using value sets from the United Kingdom (Ramos-Goni and Rivero-Arias 2011). It is sufficiently valid and reliable for use in patients with knee OA (Fransen and Edmonds 1999).

###### Comorbidities

Patients were asked to give “yes/no” responses to whether they had ever had any of the following comorbidities: osteoporosis, gout, hypertension, stroke, heart attack, heart failure, high cholesterol, diabetes, and renal, bowel, lung, or liver problems. The number of comorbidities for each patient was assessed and divided into 2 categories for the analysis (no comorbidities vs. ≥ 1 comorbidity).

##### Postoperative pain expectation

Patients responded to the following question: “Overall, how much do you expect that pain in your knee will interfere with your life 12 months after surgery?” by selecting an option from a 5-point Likert scale (Not at all, Mildly, Moderately, Severely, Extremely). Due to few patients expecting severe/extreme (n = 6, 0.8%) or moderate (n = 53, 7.2%) pain, this variable was dichotomized to “not at all” vs. “mildly to extremely”.

### Statistics

To determine differences between patient characteristics in UKA and TKA groups, unequal t-tests, chi-squared tests or Mann–Whitney U-tests were performed, as appropriate. Linearity of the effect between continuous variables with return to desired activity was assessed using fractional polynomials. This is a flexible parametric method that models the relationship between exposure and outcome, enabling findings to be interpreted by readers without expertise in statistics (Royston et al. 1999). Interactions between procedure type and all other potential predictors were evaluated. Missing data were assumed to be missing at random. Multiple imputation using chained equations was performed and 50 datasets were generated on the total dataset (including statistically significant (p < 0.05) interactions) and on both UKA and TKA subgroups, separately. To assess whether the imputed data accurately reflected the raw data, distribution and associations between imputed variables and outcome were compared between the raw and imputed data. Since similar results were observed between raw and imputed datasets, we considered that imputation was appropriate. Generalized linear models (GLM) using a log link, Poisson family, and robust error variances (Zou 2004) were applied to assess the association of potential predictors on outcome. Risk ratios (RR) and the corresponding 95% confidence intervals (CI) were calculated. All analyses were completed using Stata/IC 14.1 (StataCorp, College Station TX, USA).

#### Ethics, funding, and potential conflict of interest

Ethics approval for COASt was obtained: Oxford REC A (Ethics Reference: 10/H0604/91). This article presents independent research commissioned by the National Institute for Health Research (NIHR) under its Programme Grants for Applied Research funding scheme (RP-PG-0407-10064). Prof Arden and Dr Filbay are supported by the Arthritis Research UK Centre for Sport, Exercise and Osteoarthritis (grant reference 21595). The views of the author(s) expressed in this article do not necessarily represent those of the National Health Service, the NIHR, or the Department of Health. NKA is a consultant for Freshfields Bruckhaus Deringer and has received honorariums from Bioventus, Flexion, Regeneron, and grants from Bioiberica and Merck outside the submitted work. ADH, MTSS, and SRF declare no conflict of interest.

## Results

### Patient characteristics (Table 1)

Patients (55% women) were a mean 69 (SD 9) years old at the time of surgery and had a mean BMI of 30 (SD 5). 86% of patients reported ≥1 comorbidity. 34% of all patients did not return to desired activity 12 months after knee surgery.

**Table 1.** Patient characteristics at baseline and 12 months post-arthroplasty

	All patients	Missing	UKA	TKA	
	(n = 995)	data	(n = 420)	(n = 575)	p-value a
Baseline characteristics					
Age at operation^b^	69 (9)	0 (0%)	67 (10)	70 (9)	< 0.001
Female sex^c^	549 (55%)	0 (0%)	220 (52%)	329 (57%)	0.1
BMI^b^	30 (5)	5 (0.5%)	30 (5)	31 (6)	0.006
Education level^c^		159 (16%)			
Did not complete GCSE or above	385 (39%)		144 (42%)	241 (49%)	0.04
Completed GCSE or above	451 (45%)		201 (58%)	250 (51%)	
Baseline EQ-5D^d^	0.62 (0.16–0.69)	106 (11%)	0.62 (0.16–0.69)	0.59 (0.16–0.69)	0.05
Postoperative pain expectation^c^**^,e^**		273 (27%)			
No pain expected	389 (39%)		172 (56%)	217 (52%)	0.3
Mild to extreme pain expected	333 (34%)		133 (44%)	200 (48%)	
Baseline UCLA Activity Score^d^	4 (3–5)	283 (28%)	4 (3–5)	4 (3–5)	0.1
No reported comorbidities^c^**^, f^**	140 (14%)	245 (25%)	75 (25%)	65 (15%)	< 0.001
Baseline OKS^b^	20.4 (7.7)	79 (8%)	21.6 (7.6)	19.5 (7.7)	< 0.001
12 months post-surgery					
Non-return to desired activity^c^**^,g^**	341 (34%)	0 (0%)	105 (25%)	236 (41%)	<0.001

a p-values for differences between UKA and TKA groups, assessed using unequal t-tests, chi-squared tests or Mann–Whitney U-tests as appropriate.

b Mean (SD)

c Number (%)

d Median (IQR)

**^e^**Postoperative pain expectation: assessed preoperatively using a 5-point Likert scale (Not at all, Mildly, Moderately, Severely, Extremely) in response to the question: “Overall, how much do you expect that pain in your knee will interfere with your life 12 months after surgery?”

f No reported comorbidities: the number of patients who had not been diagnosed with any of the following comorbidities (compared with a diagnosis of 1 or more of these comorbidities): osteoporosis, gout, hypertension, stroke, heart attack, heart failure, high cholesterol, diabetes, renal, bowel, lung, and liver problems.

g Non-return to desired activity: Proportion of patients that responded “No” to the following question at 12-month follow-up: “Have you been able to return to the activity (or activities) that your knee stopped you from doing 12 months ago?”

UKA: unicompartmental knee arthroplasty; TKA: Total knee arthroplasty; BMI: body mass index; GCSE: General Certificate of Secondary Education; EQ-5D: EuroQol 5 dimensions questionnaire; UCLA: University of California, Los Angeles; OKS: Oxford Knee Score.

UKA was performed on 42% (n = 420), and TKA on 58% (n = 575) of patients. Patients who had TKA were older (70 (9) vs. 67 (10), p < 0.001), had a greater mean BMI (31 (6) vs. 30 (5), p = 0.006) and a greater percentage did not complete GCSE or above (49% vs. 42%, p = 0.04) compared with UKA patients. Patients who had UKA reported better baseline median EQ-5D scores (0.62, IQR (0.16–0.69) vs. 0.59 (0.16–0.69), p = 0.05), better baseline mean OKS scores (22 (7.6) vs. 20 (7.7), p < 0.001), and a greater percentage reported no comorbidities (25% vs. 15%, p < 0.001) compared with TKA patients.

#### Return to desired activity 12 months following UKA vs. TKA (Table 1)

The percentage of patients that did not return to desired activity 12 months following surgery was greater following TKA than UKA (41% vs. 25%, p < 0.001). The most common activities that patients wished to return to were similar between UKA and TKA patients; the 4 most common activities were walking (UKA 58%; TKA 57%), gardening (14%; 16%), cycling (8%; 9%), and swimming (9%; 7%).

#### Predictors of return to desired activity 12 months following knee arthroplasty (Table 2)

A 1-unit greater baseline OKS score was associated with a 3% lower risk of not returning to desired activity (RR 0.97 (0.95–0.99)). Patients who expected some degree of pain interference with life 12 months post-arthroplasty had a 1.3 (1.1–1.7) times greater risk of not returning to desired activity compared with patients who expected no pain interference. TKA was associated with a higher risk of not returning to desired activity compared with UKA (1.5 (1.2–1.8)).

**Table 2.** Multivariable analysis reporting risk ratios for non-return to desired activity following a generalized linear model for all, UKA, and TKA patients

	All patients (n = 995)		UKA (n = 420)		TKA (n = 575)	
Predictors	RR (95% CI)	p-value	RR (95% CI)	p-value	RR (95% CI)	p-value
Age	1.00 (0.99–1.01)	1.0	0.99 (0.97–1.01)	0.2	1.01 (1.00–1.02)	0.09
Sex^a^	1.15 (0.96–1.38)	0.1	1.43 (0.99–2.05)	0.06	1.04 (0.85–1.27)	0.7
BMI	1.00 (0.98–1.01)	0.7	1.04 (1.01–1.08)	0.006	0.99 (0.97–1.00)	0.1
Baseline OKS	0.97 (0.95–0.99)	0.001	0.96 (0.93–0.99)	0.02	0.98 (0.96–1.00)	0.04
Baseline UCLA-AS	1.02 (0.95–1.10)	0.6	1.07 (0.94–1.23)	0.3	1.00 (0.92–1.09)	1.0
Baseline EQ-5D Score	0.71 (0.47–1.07)	0.1	0.91 (0.43–1.92)	0.8	0.53 (0.33–0.85)	0.008
Pain expectation^b^	1.34 (1.09–1.65)	0.005	1.86 (1.24–2.78)	0.003	1.15 (0.92–1.45)	0.2
Education^c^	0.94 (0.78–1.13)	0.5	1.08 (0.76–1.52)	0.7	0.95 (0.76–1.18)	0.6
Comorbidities^d^	1.04 (0.98–1.11)	0.2	1.08 (0.95–1.22)	0.3	1.03 (0.95–1.11)	0.5
Procedure^e^	1.47 (1.21–1.78)	< 0.001				

All variables were included in these multivariable analyses, except for “Procedure” in the UKA and TKA analyses.

Outcome coded as Returned-to-desired-activity =0, and Did-not-return-to-desired-activity =1

a Female =1 (compared with Male =0).

b Pain expectation: “Some =1” (mildly, moderately, severe or extremely) compared with “none =0” (not at all) (preoperative response to the following question: “Overall, how much do you expect that pain in your knee will interfere with your life 12 months after surgery?”).

c “Completed GCSE or above” = 1 (compared with “did not complete GCSE or above” = 0).

d ≥ 1 comorbidity =1 (compared with no comorbidities =0).

e TKA =1 (compared with UKA =0).

For abbreviations, see Table1

#### Predictors of return to desired activity 12 months following UKA vs. TKA (Table 2)

***UKA*** — For every 1-unit greater BMI, patients who underwent a UKA had a 4% greater risk of not returning to desired activity (RR 1.04 (1.01–1.08)). UKA patients who expected some degree of postoperative pain interference had a 1.9 (1.2–2.8) times greater risk of not returning to desired activity compared with UKA patients who expected no postoperative pain interference. With every 1-unit better baseline OKS, the risk of not returning to desired activity was 4% lower (0.96 (0.93–0.99)) following UKA. Baseline EQ-5D did not predict return to desired activity following UKA.

***TKA*** — A 1-unit better baseline OKS corresponded to a 2% lower risk of not returning to desired activity following TKA (0.98 (0.96–1.00)). Better EQ-5D values before undergoing TKA were associated with a lower risk of not returning to desired activity (0.53 (0.33–0.85)). BMI, expectations, and sex did not predict return to desired activity following TKA.

## Discussion

A greater proportion of UKA patients returned to desired activity 12 months after arthroplasty, compared with TKA patients. For both UKA and TKA, the most common desired activities patients wished to return to were walking, gardening, cycling, and swimming. TKA patients had a 1.5 times greater risk of not returning to desired activity compared with UKA patients. Similarities and differences were found in predictors of non-return to desired activity between UKA and TKA patients. Better baseline OKS predicted better outcome following both UKA and TKA. Higher BMI and worse expectations only predicted non-return to desired activity after UKA. On the other hand, worse baseline EQ-5D scores predicted non-return to desired activity following TKA, but not following UKA. Age, preoperative activity level, education level, and comorbidities were not associated with return to desired activity.

We found that, irrespective of arthroplasty procedure, patients who had less preoperative knee pain and better function were more likely to return to desired activity. Less preoperative knee pain and better function have also been found to be associated with postoperative satisfaction and better OKS after both UKA and TKA (Munk et al. 2011, Judge et al. 2012, Sanchez-Santos et al. 2018). Preoperative exercise is one strategy that may be effective in reducing knee pain and improving function prior to knee arthroplasty (Wallis and Taylor 2011). Patients on the waiting list for knee arthroplasty who report a large degree of knee impairment may benefit from targeted management to improve knee pain and function.

We found that a greater BMI was associated with not returning to desired activity after UKA. This is in line with Williams et al. (2012) who reported an association between greater preoperative BMI and worse activity outcomes 12 months after UKA. On the other hand, other studies found BMI did not predict revision surgery, postoperative knee pain, function, or satisfaction following UKA (Liddle et al. 2014, Burnett et al. 2014, Zuiderbaan et al. 2016). Thus, the relationship between higher preoperative BMI and post-UKA activity limitations may be explained by factors other than the clinical status of the knee (such as motivation, deconditioning, knee confidence), although this was not specifically explored in this study. Weight management in obese patients awaiting UKA may improve a patient’s ability to return to desired activity. This may be a valuable area for future research, considering there is insufficient evidence to determine the effectiveness of short-term non-pharmacological, non-surgical weight management interventions on patient outcomes following knee arthroplasty (Lui et al. 2015).

A previous study in the COASt cohort performed an in-depth analysis of the relationship between pain expectations and outcome (Filbay et al. 2018). Patients who expected moderate to extreme pain interference had greater odds of being dissatisfied and not achieving a meaningful improvement on the OKS compared with those who expected no pain interference, irrespective of arthroplasty procedure (Filbay et al. 2018). However, the odds of a poor outcome (not returning to desired activity, postoperative dissatisfaction, not achieving minimally important change in OKS) in patients expecting moderate to severe postoperative pain were higher following UKA compared with TKA (Filbay et al. 2018). Further research is needed to explore and compare patient expectations between UKA and TKA procedures. Considering expectations are potentially modifiable, targeted education for preoperative patients undergoing UKA with poor expectations has potential to improve postoperative outcome (Mancuso et al. 2008, McDonald et al. 2014).

Concordant with our findings, there is support for baseline EQ-5D as a predictor of TKA outcomes (Judge et al. 2012). Worse preoperative general health and the presence of anxiety or depression (assessed in the EQ-5D measure) have been found to predict less improvement in OKS following TKA (Baker et al. 2012, Hanusch et al. 2014). However, there is a need to further investigate the relationship between baseline EQ-5D scores and postoperative outcome in UKA populations.

By comparing predictors of return to desired activity following UKA and TKA, our results both support and refute elements of traditional selection criteria. Our findings substantiate Kozinn and Scott (1989) who recommended that patients with a BMI below the obese category, and those with less preoperative knee pain, may be most likely to have a favorable outcome following UKA. However, in contrast to Kozinn and Scott, we found no evidence to suggest selection should be based on patient age or preoperative activity level. Other studies have also reported no association between age and other postoperative outcomes (revision rate, knee pain, stiffness or function) (Burnett et al. 2014, Lewis et al. 2015, Zuiderbaan et al. 2016, Alattas et al. 2017). Notably, traditional guidelines were not designed with reference to return to desired activity. Considering that more recent recommendations are similar to the Kozinn and Scott framework (National Imaging Associates Inc. 2015, Quinn et al. 2017), patient selection criteria for UKA vs. TKA procedures should be updated based on current evidence, with a greater focus on patient-centered outcomes. . In addition to informing treatment selection for patients with unicompartmental knee OA, our findings highlight patients at risk of poor outcome who may benefit from targeted preoperative intervention (e.g. preoperative rehabilitation for patients with severe knee pain and poor function, weight-loss strategies for overweight patients, and education for patients expecting a poor surgical outcome).

Strengths of this study include the large sample size, comprehensive baseline data enabling control of confounders (a common limitation in previous studies), and the large number of patients who had UKA. The most common activities that UKA and TKA patients desired to return to were comparable. However, further analyses in UKA and TKA sub-groups of patients who expect to return to higher or lower levels of activity following surgery may be a fruitful area for future research. Due to limitations inherent to non-randomized studies, preoperatively UKA patients were younger, had better OKS, lower BMI, and better EQ-5D scores than TKA patients. Since these differences were accounted for in multivariable analysis, they are unlikely to explain the observed between-group differences in return to desired activity. Patients lost to follow-up represent a potential for bias and reduce the representativeness of the sample to all patients having knee arthroplasty. We did not have information regarding which knee compartments were affected by arthritis. It is possible this was related to the observed differences in return to desired activity between procedures.

In summary, we found that TKA patients were less likely to return to desired activity than UKA patients. UKA patients with a high BMI, worse preoperative pain/function, and patients with worse pain expectations had a greater risk of not returning to desired activity, compared with other UKA patients. TKA patients with worse preoperative pain/function and those with a worse preoperative health status were at greater risk of not returning to desired activity compared with other TKA patients. This information may assist in identifying patients who may benefit from targeted preoperative intervention to improve surgical prognosis.

All authors conceived and designed this analysis. ADH wrote the first draft of the manuscript. NKA contributed to all phases of design, obtaining funding and data collection for the COASt cohort. ADH, MTSS, and SRF analyzed and interpreted the data, edited and revised the manuscript.

The authors would like to thank the individuals who participated in the COASt Study, the National Institute for Health Research (NIHR), and the Oxford Medical Sciences Division for their funding support to the study, and the rest of the COASt team for their time and dedication: D Altman, D Beard, A Carr, C Cooper, D Culliford, T Griffin, K Javaid, J Latham, D Murray, R Pinedo-Villanueva, A Price, and D Prieto-Alhambra.

*Acta* thanks Nanne Kort and Paul Kuijer for help with peer review of this study.
